# A biomaterial-silicon junction for photodetection

**DOI:** 10.1016/j.mtbio.2023.100642

**Published:** 2023-04-24

**Authors:** Narendar Gogurla, Abdul Wahab, Sunghwan Kim

**Affiliations:** a Department of Energy Systems Research, Ajou University, Suwon, 16499, South Korea; b Department of Biomedical Engineering & Department of Electronic Engineering, Hanyang University, Seoul, 04763, South Korea

**Keywords:** Melanin, Silk hydrogel, Junction, Photodetector, Image sensor

## Abstract

Bio-integrated optoelectronics can be interfaced with biological tissues, thereby offering opportunities for clinical diagnosis and therapy. However, finding a suitable biomaterial-based semiconductor to interface with electronics is still challenging. In this study, a semiconducting layer is assembled comprising a silk protein hydrogel and melanin nanoparticles (NPs). The silk protein hydrogel provides a water-rich environment for the melanin NPs that maximizes their ionic conductivity and bio-friendliness. An efficient photodetector is produced by forming a junction between melanin NP-silk and a *p*-type Si (*p*-Si) semiconductor. The observed charge accumulation/transport behavior at the melanin NP-silk/*p*-Si junction is associated with the ionic conductive state of the melanin NP-silk composite. The melanin NP-silk semiconducting layer is printed as an array on an Si substrate. The photodetector array exhibits uniform photo-response to illumination at various wavelengths, thus providing broadband photodetection. Efficient charge transfer between melanin NP-silk and Si provides fast photo-switching with rise and decay constants of 0.44 ​s and 0.19 ​s, respectively. The photodetector with a biotic interface comprising an Ag nanowire-incorporated silk layer as the top contact can operate when underneath biological tissue. The photo-responsive biomaterial-Si semiconductor junction using light as a stimulus offers a bio-friendly and versatile platform for artificial electronic skin/tissue.

## Highlights

• Fully biomaterial-based and photo-responsive semiconductor assembled by silk protein and melanin nanoparticles.
• A semiconductor junction utilized by silk-melanin bio-semiconductor and silicon, and its photodetection application.
• A broadband photodetection covering the UV to near-IR spectral region.
• Fast photo-switching due to the seamless interface between bio-semiconductor and silicon.
• Photodetection of the light signal through an animal tissue on the bio-photodetector.

## Graphical abstract

A fully biomaterial-based semiconductor and its use for image sensing has been realized. Silk protein and melanin nanoparticles form the semiconductor junction with silicon and show the photocurrent by light stimulation, resulting in photodetection applications.

**Figure:**
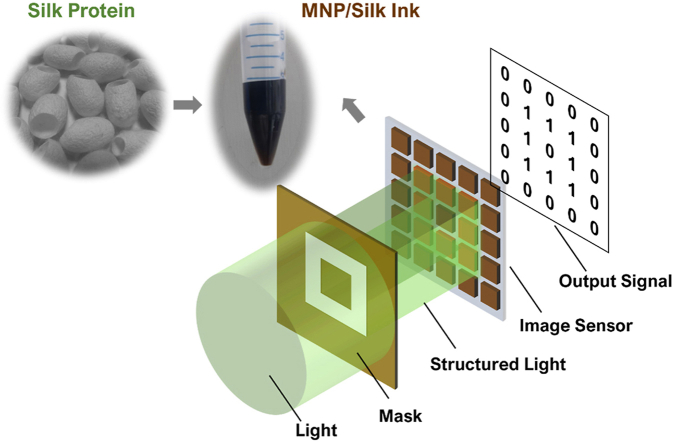

## 1 Introduction

Bio-integrated optoelectronics using light-matter interaction, such as diode-based light emitters and light detectors, have recently emerged as promising candidates for skin- and tissue-implantable devices that can enable a wide range of applications, including artificial retinas, healthcare monitoring, and photo-responsive components in soft-robots [[1], [2], [3], [4], [5]]. For a biotic-abiotic interface, integrating biomaterials with inorganic semiconductors has been widely investigated to achieve efficient bio-integrated electronic devices that can interface with skin and tissue [1,6]. A few attempts to integrate biomaterials with semiconductors such as Si [[7], [8], [9]], zinc oxide (ZnO) [8,10], and titanium dioxide (TiO_2_) [11] to realize electronic and optoelectronic devices have been reported. Kang et al*.* [12] produced a bioresorbable electronic sensor using Si for biomedical applications. Dagdeviren et al. [13] fabricated transient electronics based on ZnO and silk for use in resorbable biomedical implants. Son et al. [14] developed a wearable electronic patch utilized by a TiO_2_ nanomembrane and Au nanoparticles (NPs) on an elastomeric hydrocolloid skin patch for the storage of sensor activity data. Semiconducting properties are essential when designing bio-integrated electronic and optoelectronic devices. Among the semiconducting materials, Si has already been utilized in the electronic and photonic industry owing to its excellent electrical and optical properties. However, due to its narrow band gap of 1.12 ​eV, it does not show a broadband photodetection response and is inefficient in the ultraviolet (UV) and blue light ranges, thereby making it unsuitable for photodetection [15,16]. Making a junction with other materials such as molybdenum(IV) sulfide (MoS_2_) is key for broadband photodetection by Si-based photodetectors.^[15]^ In addition, for bioelectronic applications that require a seamless biotic-abiotic interface, a bio-friendly semiconducting material platform with excellent UV and visible (Vis) absorption and the ability to form a junction is highly desirable.

**Fig. 1:**
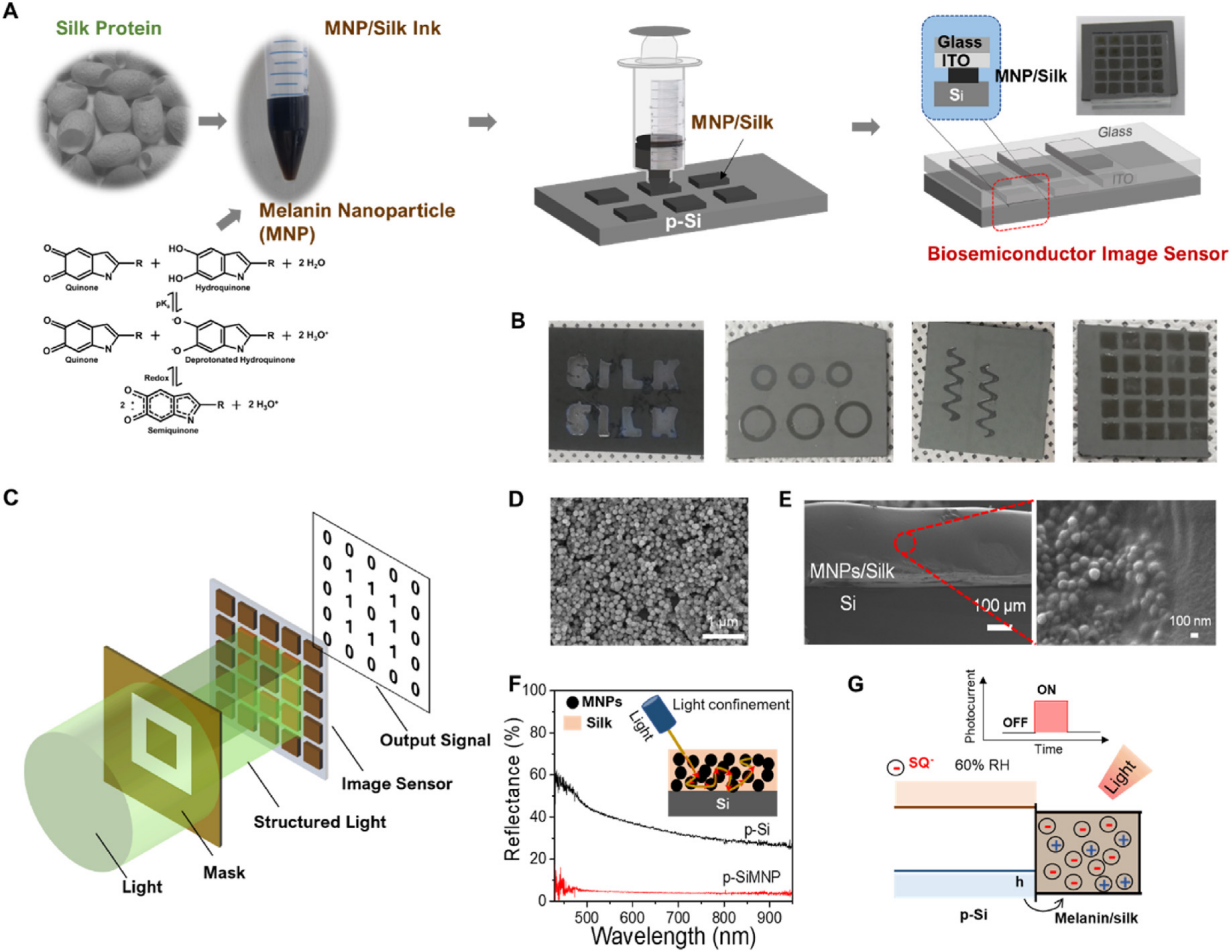
The 3D printing process for the melanin NP-silk hydrogel/Si photodetector arrays. (A) The device fabrication process including the 3D-printing of an array on Si followed by its attachment to ITO/glass. (B) Optical images of printed structures of melanin NP-silk hydrogel on Si. (C) A schematic of the concept of applying the photodetector array for image sensing. (D) An SEM image of as-prepared melanin NPs. (E) A cross-sectional SEM image of melanin NP-silk/Si layers. The left panel is a magnified view of melanin NPs embedded in a silk layer. (F) Reflectance spectra of *p-*Si and p-Si/melanin NPs. The inset is a schematic showing light confinement by the melanin NPs. (G) The mechanism involved in generating photocurrent by the device.

Melanin is a class of biological pigments with unique and interesting properties, including broadband UV–Vis absorption, photo-protection, free radical scavenging, and a mixed protonic/electronic nature [[17], [18], [19], [20], [21], [22], [23]]. In general, the molecular structures of melanin are 5,6-dihydroxyindole (DHI) and 5,6-dihydroxyindole 2-carboxyindole (DHICA), along with their redox states hydroquinone, quinone imine (QI), indole quinone (IQ), and semiquinone (SQ) (Fig. 1A). The conduction of free radicals generated in hydrated melanin makes it suitable as a semiconducting biomaterial. The semiconducting properties of melanin were first uncovered by McGinnes et al. [24,25] to explain its electrical properties. Owing to its hydration-dependent protonic conductivity, melanin could emerge as a model semiconducting biomaterial that can form junctions with other semiconductors. Since hydration is the critical factor for revealing the semiconducting properties of melanin, its combination with a hydrogel provides a suitable platform for melanin-based semiconductor devices. Silk protein has been recognized as a versatile material for bioelectronics and biophotonics due to its biocompatibility, biodegradability, flexibility in thin film form, and functionalizability by blending or doping it with other materials [[26], [27], [28], [29], [30], [31], [32]]. In our previous work, we investigated the electrical and optical properties of a semiconducting biomaterial comprising silk to form a hydrogel matrix for melanin in the presence of water [26,27].

Herein, we report a bioelectronic photodetector created by forming a junction of melanin NPs incorporated in a silk hydrogel with Si to provide an effective biomaterial/inorganic hybrid diode photodetector. The electrical properties of the junctions of the melanin NP-silk hydrogel layer with *p*-Si or *n*-Si enable insight into mixed ionic/electronic transport mechanisms. Different charge accumulation and transport mechanisms at the melanin NP-silk/Si interface were observed, which are mainly attributed to the different charge carriers in *p*-Si (holes) and *n*-Si (electrons) and the ionic conductive state of the melanin NP-silk layer. We discovered that melanin NP-silk/*p*-Si is suitable for photodetection applications. The melanin NP-silk/*p*-Si photodetector array exhibited a broadband photodetection response (from UV to near-infrared (NIR)) to light illumination. Moreover, the relative humidity affected the electrical current measurements because increasing the humidity and light illumination increases the number of ionic free radicals in the melanin NP-silk layer that facilitate hole transfer from *p*-Si to the melanin NPs. The seamless interface between melanin NP-silk and *p*-Si enabled efficient charge transfer between them, and thus we achieved fast photo-switching with rise and decay values of 0.44 ​s and 0.19 ​s, respectively. A transparent biocompatible top contact produced by incorporating Ag nanowires (AgNWs) into a silk protein layer was used instead of an indium tin oxide (ITO)/glass electrode with the melanin NP-silk/*p*-Si photodetector. The AgNW-silk electrode provided a biocompatible interface with pigskin that made photodetection possible via light illuminated through the skin.

## 2 Results and discussions

Fig. 1A depicts the fabrication process for the melanin NP-silk-hydrogel-based bio-photodetector array on a *p*-Si substrate. Briefly, photoconductive bioink was prepared by mixing melanin NPs with silk protein solution and then printed on the *p*-Si substrate. Thereafter, a transparent electrode comprising ITO/glass or an AgNW layer was placed on the patterned melanin NP-silk photodetector array. We utilized the self-healing ability of the silk hydrogel to fabricate the ITO/melanin NP-silk/*p*-Si photodetector as strongly adhered layers with seamless interfaces between them. It was possible to print various structures (e.g., the letters in ‘SILK,’ circles, serpentines, squares, etc.) using the melanin NP-silk bioink at a resolution of 0.2 ​mm (Fig. 1B). We have attempted to produce a photodetector array using various sizes (ranging from 100 to 200 ​nm) and concentrations (1.5, 2.2, and 3 ​wt%) of MNPs in the silk hydrogel during the printing process. Our results show that melanin NPs with a size of 120 ​nm and a concentration of 2.2 ​wt% are well-dispersed in silk hydrogel and are the most suitable for fabricating clear pixels on the Si substrate compared to other combinations of sizes and concentrations of melanin NPs (Fig. 1B and Fig. S1). At higher concentration (3 ​wt%), the printing is not possible due to high viscosity of bioink (i.e., ink is not coming from the nozzle as shown in Fig. S1). The schematic illustration in Fig. 1C illustrates how the melanin NP-silk can be used to create biomaterial-based photo-responsive pixels to mimic retinal cells. The scanning electron microscopy (SEM) image of the as-prepared melanin NPs in Fig. 1D reveals their spherical shape with an average diameter of ∼120 ​nm. A cross-sectional SEM view of the as-printed bio-photodetection layer on an Si substrate is shown in Fig. 1E. The thickness of the melanin NP-silk layer is found to be ∼300 ​μm, and it also indicates that melanin NPs were embedded in the silk protein matrix, which can be clearly seen in the magnified SEM image in Fig. 1E. Fig. 1f shows a cross-sectional SEM image of the whole device structure, indicating a thickness reduction of the melanin NP-silk layer to ∼180 ​nm after attaching it to the ITO/glass substrate due to the applied pressure (0.56 ​kPa) required for bonding. We measured the reflectance of the Si substrates with and without the melanin NP-silk layer (Fig. 1F). The melanin NP-silk printed sample showed very low reflectance in the broad visual-IR spectral range (400–1000 ​nm), whereas pristine Si exhibited relatively high reflectance in the range of 30%–60%. To gain a deeper understanding of the absorption properties of melanin NPs and silk hydrogel, we measured their absorption spectra as well as that of their composite material. The results showed that the silk hydrogel layer exhibited negligible absorption in the range from UV to near IR, while the melanin NPs exhibited featureless absorption in the UV range (Fig. S2). This is related to the broadband photo-absorption by melanin and the enhancement in absorption due to light scattering by the melanin NPs making the optical path longer. Fig. 1G shows the photo-response mechanism of the melanin NP-silk photodetector. Briefly, light illumination generates free radicals that facilitate the capturing of holes from *p*-Si at the junction, which increments the current in the device.

**Fig. 2:**
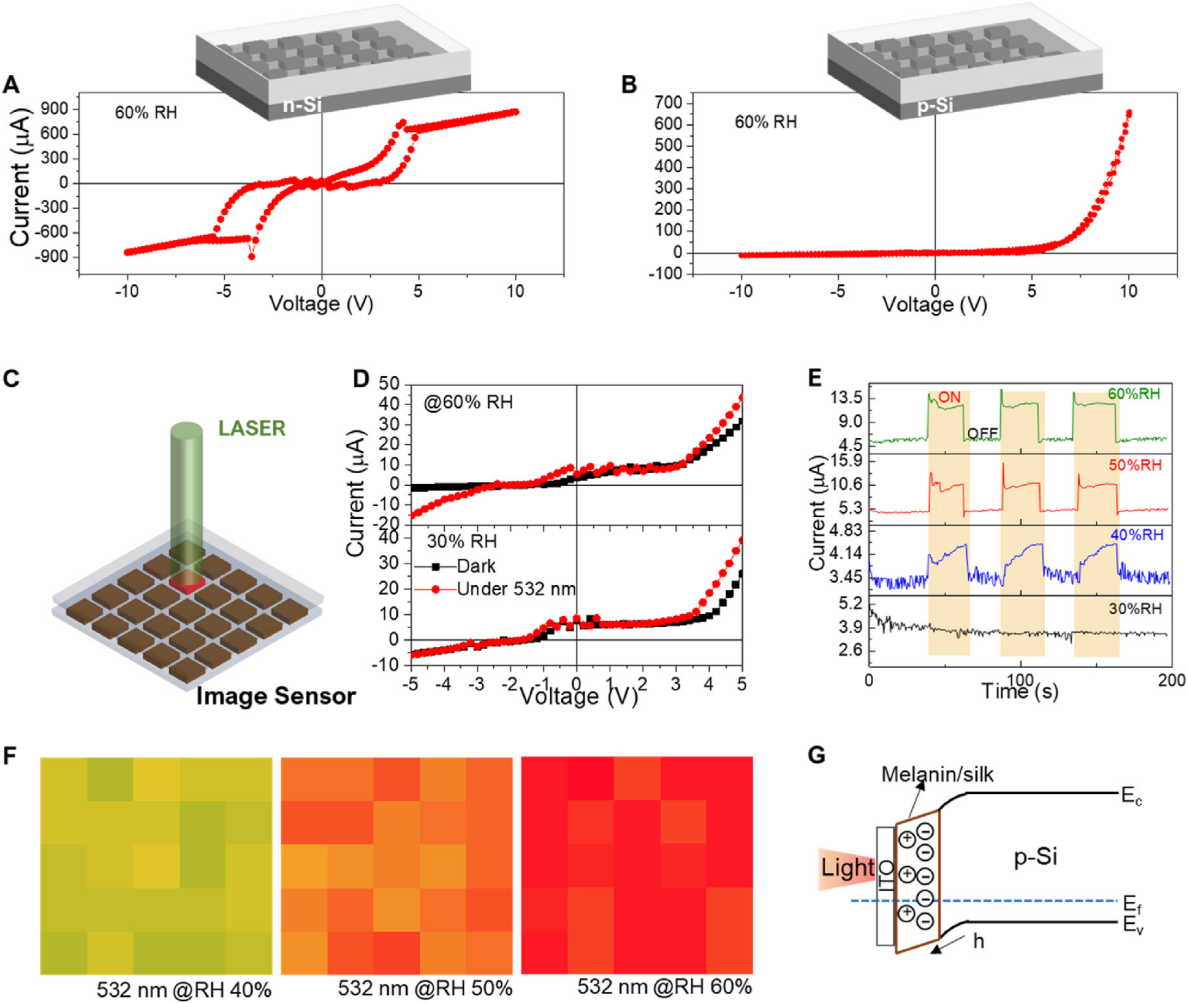
Electrical and photodetection properties of the ITO/NP-silk hydrogel/Si photodetector array. I–V characteristics of the photodetector arrays fabricated on (A) *n*-Si and (B) *p*-Si. (C) A schematic representation of photodetection measurements using the device. (D) The *I–V* characteristics of the device containing *p-*Si at various humidity levels and under dark or 532 ​nm illumination conditions. (E) The transient photocurrent of the device at various humidity levels. (F) Photocurrent contour plots of the device under 532 ​nm light and various humidity levels. (G) The band structure and charge transfer process in the device.

To investigate the electrical properties of melanin NP-silk composites with various concentrations of NPs, we measured their electrical conductivities at a relative humidity (RH) of 60% (Fig. S3a); the conductivity increased with increasing melanin NP concentration due to more SQ^−^ free radicals being generated. In addition, optical transmittance spectra indicate a decrement in transparency in the vis-IR range with increasing melanin NP concentration (Fig. S3b). In the visible region (400–700 ​nm), absorption by the melanin NP-silk layer decreased as the wavelength was increased. However, at a concentration of <4.5 ​wt% melanin NPs, the optical transparency became negligible over the whole wavelength region, indicating that all of the incident light was fully absorbed by the melanin NP-silk layer that was only 180 ​nm thick. We chose 2.2 ​wt% as the optimal concentration of melanin NPs in the silk hydrogel and used this ratio to print a 5 ​× ​5-pixel array on Si and utilized it as a semiconducting layer to make a junction with Si. To investigate the effect of the charge carrier type of Si on the photodetection characteristics, the melanin NP-silk photodetector arrays were printed on *n*-type and *p*-type Si. Fig. 2A and B shows the measured current-voltage (I–V) curves for the devices. The hysteresis behavior of the *n*-type Si-based photodetector indicates a large accumulation of space charges at the melanin NP-silk/Si junction, thereby inducing capacitor behavior (Fig. 2A). The accumulation of space charges at the melanin NP-silk/Si junction can be clearly understood from the band structure of the device (Fig. S4). In the presence of light, the photogenerated charges further accumulate at the junction, leading to capacitor-like behavior in the device, which may not be ideal for the photodetection, as shown clearly in Fig. S4. On the other hand, in the case of *p*-type Si, the resulting device exhibited diode-like behavior instead of hysteresis, which is due to negligible accumulation of space charges at the junction that could be utilized for photodetection. The rectification ratio continued to increase sharply with increasing applied voltage, with a value of ∼63 ​at an applied voltage of 10 ​V (Fig. S5), which is comparable with those of organic-based diodes reported in the literature [33,34].

A photodetector array was fabricated on a *p*-type Si substrate and illuminated using a laser at 532 ​nm with a power density of 0.16 ​W/cm^2^ (Fig. 2C). Fig. 2D shows the I–V characteristics of a single pixel in the photodetector array under dark and light conditions at various RH levels. Along with the increment in the current (photocurrent) due to the laser illumination, the dark and photocurrent increased when the humidity was changed from 40% to 60%. Another important indicator of photodetection performance is the response time. Fig. 2E shows transient currents of the device under light ‘ON’ and ‘OFF’ conditions at various RH levels. A sharp increment in current was observed after increasing the RH from 40% to 60%. The photodetector showed a faster photo-switching speed with rise (*τ*_r_) and decay (*τ*_d_) time constants of 0.22 ​s and 0.06 ​s, respectively (Fig. S6a), in comparison to previously reported melanin NP-based optoelectronic devices.^[26,27]^ The photocurrent contour plots of the 5 ​× ​5 photodetector array in Fig. 2F indicate that they were uniform for each pixel at RH levels of 40%, 50%, and 60%; the distribution was most prominent at the highest RH (60%). It has been shown that melanin has electrical properties due to hydration-dependent ionic conductivity. In the dry form, melanin film has poor electrical conductivity and is thus mostly used as an insulating material [17,19,35,36]. In the hydrated form, a redox reaction arises in melanin due to the presence of water molecules, which generates free radicals including SQ moieties and carbon-centered radicals that induce the ionic conductivity [26,27]. Since our melanin NP-silk composite hydrogel has inherently bound water molecules, we expected a high electrical conductivity. Our findings from a previous study prove that the ionic conductivity of the melanin NP-silk hydrogel can be mainly attributed to the generation of SQ free radicals [27]. Moreover, this process becomes predominant under humid and light-illumination conditions, resulting in the photocurrent increment. For better understanding, the charge transport mechanism involved in the melanin NP-silk/*p*-Si photodetector is depicted in Fig. 2G. When the device is illuminated with shorter wavelength light (near UV range), the melanin NPs mainly absorb the light and generate negatively charged free radicals (SQ-ions). Under bias, the photogenerated SQ-ions drift towards the junction and facilitate the transfer of holes from Si to melanin, leading to the increasement of the mixed ionic/electronic conductivity (i.e., photocurrent in the device via collection of photogenerated charges at the electrodes). On the other hand, when the device is illuminated with longer wavelength light, the absorption mainly take place in the Si layer, generating photogenerated electrons in the device. For the intermediate wavelengths, the partial absorption in both layers contributes to the photo-response observed in the device. However, the free radical generation in the melanin NP-silk hydrogel layer is predominant under light illumination with shorter wavelengths due to the generation of SQ-ions, leading to the transfer of holes from Si to the junction. At the highest RH (60%), SQ-radical generation predominates further, thereby providing a stronger photo-response. If the RH exceeds 60%, such as at 80% RH, the device may become more wet, causing the pixels to spread on the Si substrate and potentially loosen the physical bonding between the ITO electrode and Si. Therefore, our device is limited to 60% RH for optimal performance as a photodetector.

**Fig. 3:**
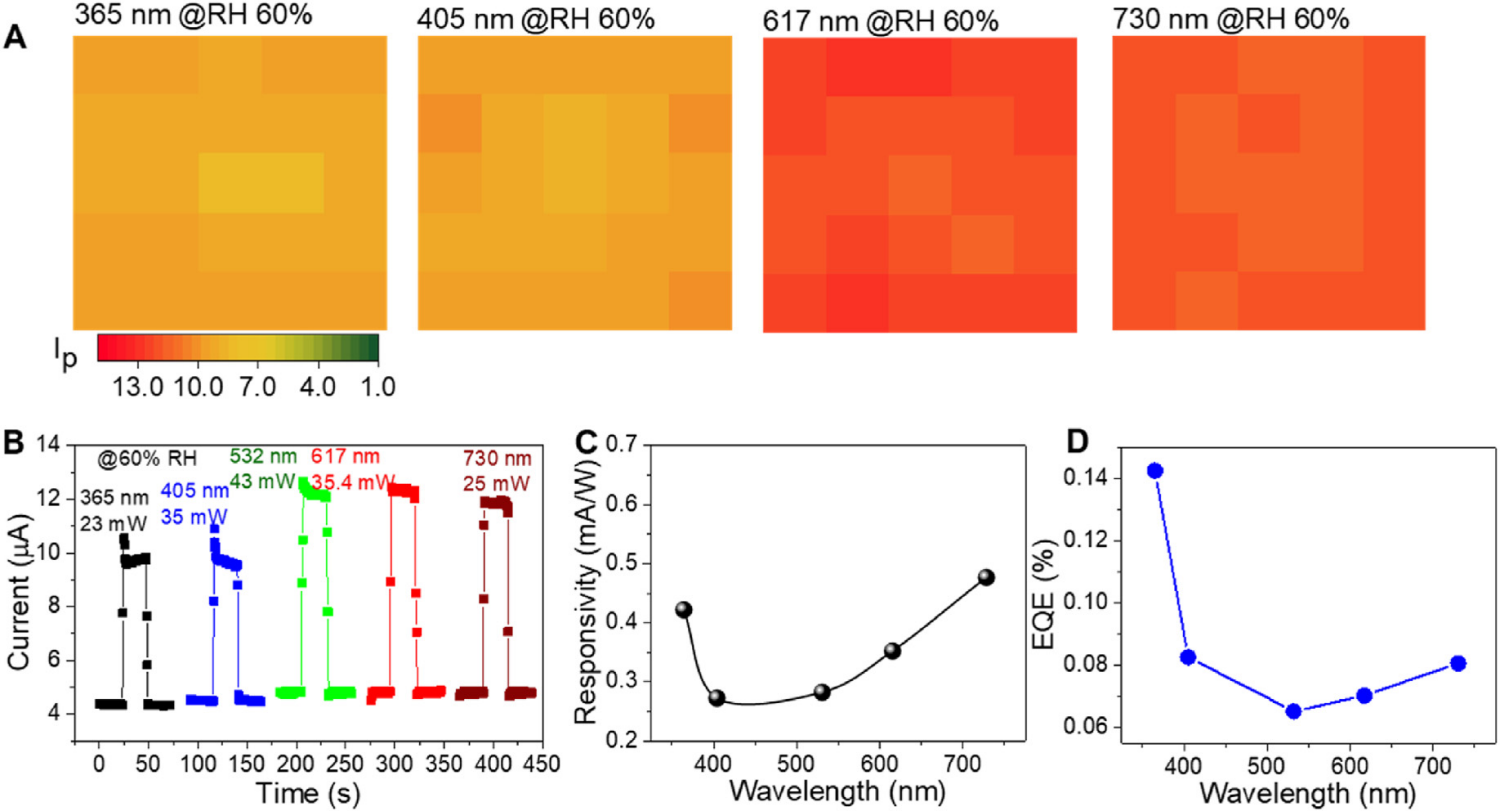
Broadband photodetection by the ITO/NP-silk hydrogel/Si photodetector array. (A) Photocurrent contour plots at various illumination wavelengths and 60% RH, (B) the transient photocurrent at various wavelengths, (C) spectral responsivity, and (D) external quantum efficiency.

Fig. 3A presents contour plots of the observed photocurrent of the 5 ​× ​5 photodetector array under different illuminating wavelengths. The device showed a notable photo-response under all of the illuminating wavelengths, thereby indicating its broadband photodetection ability from UV to NIR. Fig. 3B exhibits the transient photocurrent of the device at various illumination wavelengths; the melanin NP-silk/*p*-Si photodetector revealed a high photocurrent and fast photo-switching behavior at all of the illuminating wavelengths. The spectral responsivity can be calculated as

(1) $$R_{\lambda }=\frac{\Delta I}{PA}\text{,}$$

where Δ*I* is the difference between the photo- and dark current levels, *P* is the power density, and *A* is the area of the device. Fig. 3C shows a plot for the obtained spectral responsivity from UV to NIR of the device, thereby indicating its broadband spectral response. The spectral responsivity levels were found to be 0.42 ​mA/W at 365 ​nm and 0.47 ​mA/W at 730 ​nm due to higher absorption by the melanin NPs and *p*-Si, respectively. The absorption of Si and melanin NP-silk are more prominent in the visual-to-longer (NIR) and shorter (UV) wavelength regions, respectively (Fig. 1G and Fig. S3b). It is also obvious that melanin NP-silk is more transparent at longer wavelengths, which enables the creation of more photo-generated charge carriers in Si due to more effective absorption of the incident light. Meanwhile, the spectral responsivity is higher at shorter wavelengths due to the stronger absorption of light by the melanin NPs and at longer wavelengths (730 ​nm) due to the combined absorption of light by the melanin NPs and Si. In the wavelength range from 400 to 600 ​nm, incident light is mostly absorbed by the melanin NPs with less transmission to Si, thus providing lower spectral responsivity in the visible range in comparison to shorter (365 ​nm) and longer wavelengths (730 ​nm). The external quantum efficiency (*EQE*) of the photodetector can be calculated as

(2) $$EQE\left(\%\right)=\frac{100\times 1240\times R_{\lambda }}{\lambda }\text{,}$$

where *λ* is the wavelength of the light source. The calculated *EQE* is 0.14% at an illuminating wavelength of 365 ​nm, which is comparable to that of previously reported organic/inorganic photodetectors [37,38]. It can also be seen in Fig. 3D that the *EQE* is slightly higher for the UV region than for the visible region, which could be due to stronger absorption by the melanin NPs in the former region. Unlike wide band gap semiconductor-based photodetectors with high spectral responsivity and EQE under UV light [10], our melanin NP-silk device absorbs light exponentially in the broad wavelength range from 600 to 300 ​nm.

**Fig. 4:**
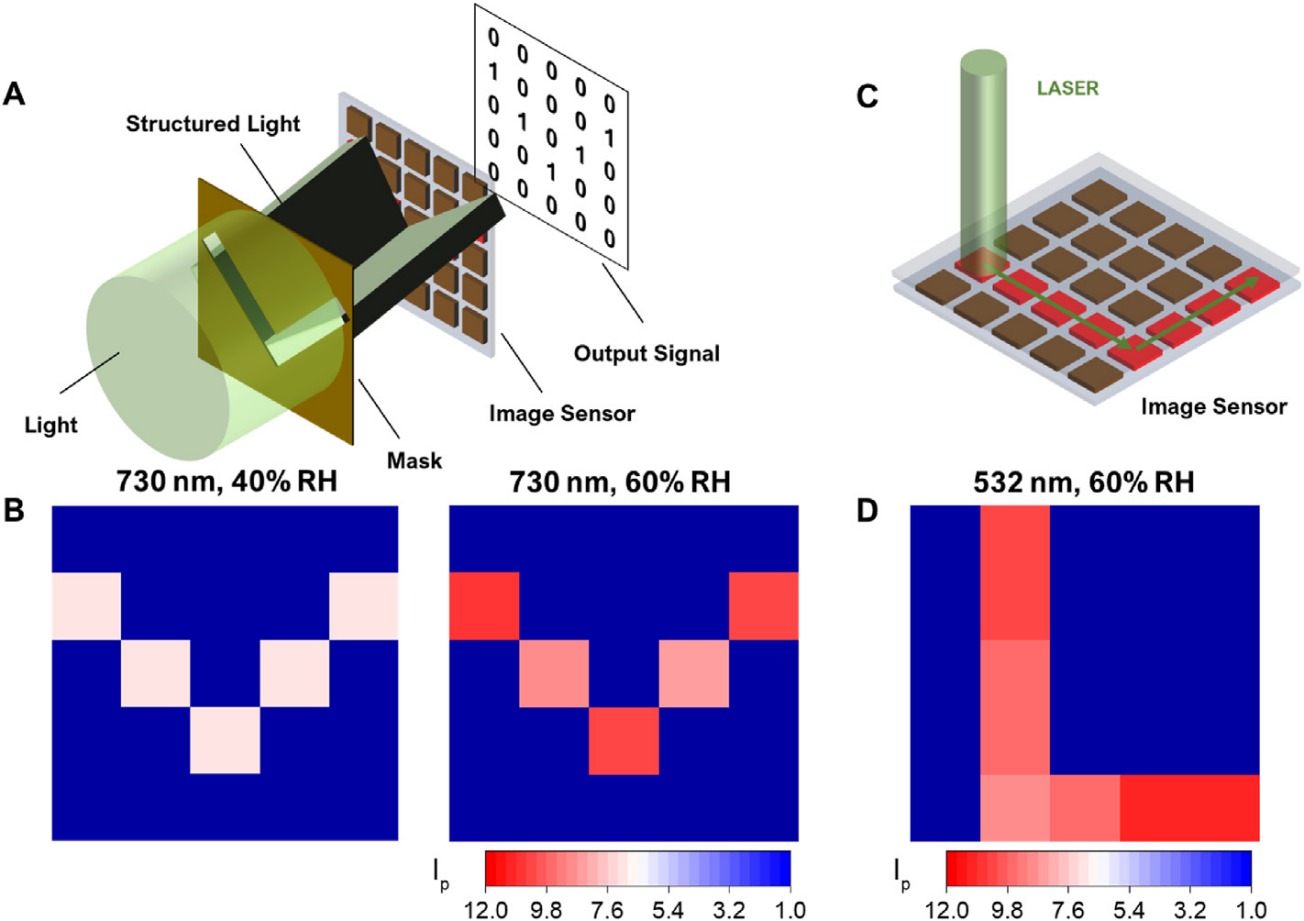
Image sensing and lettering photodetection with the ITO/NP-silk hydrogel/Si photodetector. (A) A schematic showing the image-sensing process. (B) Photocurrent contour plots under various humidity levels. (C) A schematic illustrating the process of photo-detecting letters drawn with a laser. (D) A photocurrent contour plot at 60% RH.

To investigate the image sensing capability, we obtained the photocurrent pattern from the 5 ​× ​5 photodetector array for illumination at 730 ​nm transmitted through a “V” shaped shadow mask with a letter size of 2.5 ​× ​2.5 ​cm (Fig. 4A). Fig. 4B shows the obtained photocurrent contour pattern at RH levels of 40% and 60%. Noticeable photo-responses were observed in most parts of the photodetector array, thereby providing a clear image of the “V” pattern under 730 ​nm illumination. Clear visualization of the “V” pattern was noticeable even at a low RH, indicating the good image-sensing capability of our device platform. Moreover, the movement of a laser beam could be traced by our photodetector array. Fig. 4C shows a schematic illustration of the laser-drawing application. Moreover, an “L” shape drawn with a laser beam at a power density of 0.16 ​W/cm^2^ at a frame rate of ∼0.1 fps on the array is clearly visible in the photocurrent contour plot in Fig. 4D.

**Fig. 5:**
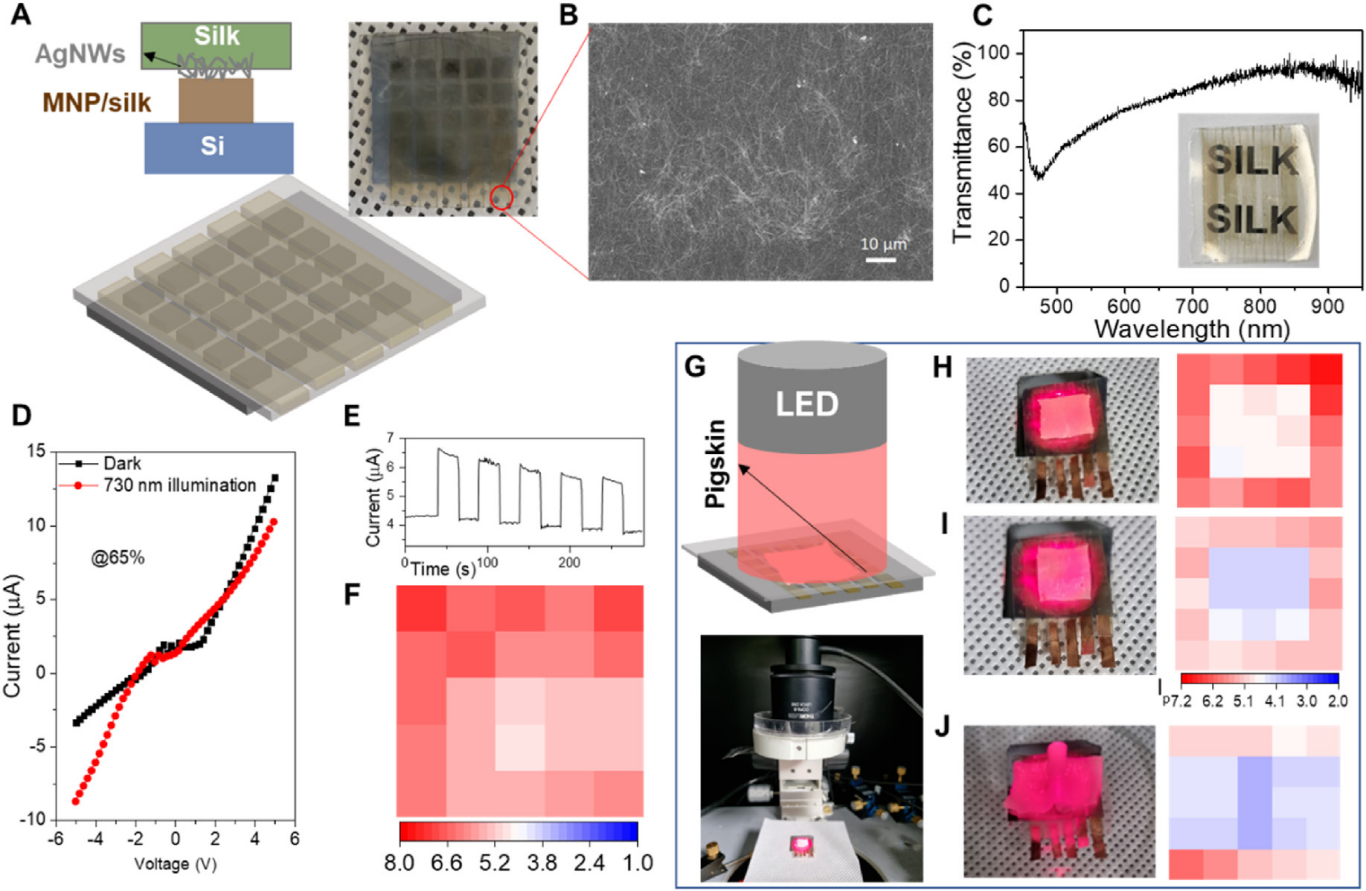
The skin-attachable biocompatible photodetector for biomedical applications. (A) A schematic representation of building the AgNW-silk membranes for the melanin NP-silk/Si photodetector array and a photograph of the resulting device. (B) An SEM image of the AgNWs impregnated in the silk layer. (C) A transmittance spectrum and a photograph of the AgNW-silk layer. D) I–V characteristics, (E) the transient current, and (F) a photocurrent contour plot of the device under 730 ​nm illumination and 65% RH conditions. (G) A schematic and a photograph of photodetection by the device through pig skin. Photocurrent contour plots for the device under 730 ​nm illumination and 65% RH conditions through pig skin layer thicknesses of (H) 0.62 ​mm and (I) 1.42 ​mm. (J) A photocurrent contour plot for photodetection by the device through folded pig skin under 730 ​nm illumination and 65% RH conditions.

To provide a biotic-abiotic interface, the top ITO/glass transparent contact was replaced with an AgNW-impregnated silk protein membrane (Fig. 5A). The top silk membrane provides a biocompatible interface for optic and electronic devices because of its bio-friendliness and similarity to human skin. To demonstrate the biocompatibility of our silk membrane, we conducted a cell culture study using HeLa cells. We used silk layers that were cleaned with either PBS or EtOH for the cell culture. After one day of cell culture, we observed that the cells had adhered well to the surface of both samples (see Fig. S7). However, the cell proliferation was significantly higher on the sample cleaned with EtOH as compared to the one cleaned with PBS. After four days of cell culture, a large number of cells had grown on the surface of both samples (Fig. S7). This clearly indicates that the silk layer is highly biocompatible and could be safely used for interfacing or even implanting in the skin. The SEM image in Fig. 5B reveals firmly embedded AgNWs in the silk protein layer, thereby enabling stable electron transport through the interconnected AgNWs. The transmission spectrum of the as-prepared AgNW/silk electrode in Fig. 5C indicates nearly 70% transmission in the Vis-to-NIR region. Fig. 5D shows *I*–*V* curves measured under dark and illumination conditions, indicating the diode-like electrical behavior and the increased photocurrent under 730 ​nm illumination. We obtained similar fast photo-switching behavior and a slight decrement in the current due to the decreased water content in the device over time (Fig. 5E). As time progresses, the water content in the silk hydrogel may decrease due to local heating resulting from multiple cycles of switching the light on and off. This could cause a slight reduction in the generation of SQ-radicals, leading to a decrease in the photo-response of the device. In addition, the bio-interfacing photodetector also showed fast photo-switching behavior (τ_r_ ​≈ ​0.44 ​s and τ_d_ ​≈ ​0.19 ​s) similar to the ITO/melanin NP-silk/Si device (Fig. S6b). Fig. 5F shows photocurrent contour plots for the biocompatible device under 730 ​nm illumination, which indicates its uniform photo-response. To show our device platform for biomedical applications, we measured the device's photodetection ability through a pig skin layer with thicknesses of 0.62 and 1.42 ​mm at 730 ​nm illumination (Fig. 5H and I, respectively). As can be observed, the device showed higher responsivity in the first biological window range for human tissues (700–980 ​nm), thereby showcasing its ability to obtain optoelectronic measurements through animal tissues and thus its biomedical applicability. Subsequently, we performed simple measurements to demonstrate the biomedical applicability of our device. The transmittance from pig skin layers at 730 ​nm with thicknesses of 0.62 and 1.42 ​mm were 30% and 15%, respectively (Fig. S8). When the thickness of the pig skin layer was increased, the photo-response of the photodetector array decreased due to variation in the pig skin layer. This further demonstrates the ability of our photodetector to monitor modulations in the thickness of pig skin. The variation in the photo-response of the photodetector array could be easily visualized by passing light through the random shape of folded pig skin with thickness variation placed on the photodetector, as shown in Fig. 5G. This photodetection ability through pig skin illustrates the utilizability of our melanin NP-silk/Si photodetector array platform for biomedical applications.

## 3 Conclusions

A bio-inspired semiconducting layer was fabricated by assembling silk protein and melanin NPs. Charge accumulation and transport at the interface between melanin NP-silk and Si depended on the charge carrier type in either *p-*Si or *n-*Si and the ionic conductive state of the bio-semiconductor layer. Photo-induced current from the bio-inspired photodetector was dependent on the RH as increasing RH increased the ionic free radical content in the melanin NP-silk and the number of hole transfers from *p*-Si to the melanin NPs. This provided efficient charge transfer at the junction, which was revealed by the fast photo-switching response (τ_r_ ​≈ ​0.44 ​s and τ_d_ ​≈ ​0.19 ​s). In addition, the photodetector showed broadband photodetection ability in the range from UV to NIR due to the complementary absorption by the melanin NPs (UV to Vis) and Si (Vis to NIR). An AgNW-incorporated silk layer provided biocompatible contact between the bio-inspired photodetector and animal tissues that enabled efficient photodetection through pigskin. The proposed biomaterial-based semiconducting platform and its photodetection applicability open new possibilities for biomedical applications and soft-robotics research.

## Credit author statement

**Narendar Gogurla**: Conceptualization, Investigation, Formal Analysis, Writing – Original Draft **Abdul Wahab**: Data Curation, Investigation, Formal Analysis **Sunghwan Kim**: Conceptualization, Writing- Original Draft, Writing – Review & Editing, Supervision, Funding Acquisition, Project Administration.

## Material and methods

***Preparation of the melanin NPs*.** First, 180 ​mg of dopamine hydrochloride (Sigma Aldrich) was dissolved in deionized (DI) water. The mixture solution was heated to 50 ​°C, after which 760 ​μL of a 1 ​M NaOH solution (Sigma Aldrich) was added for neutralization. The final solution was vigorously stirred for 5 ​h to achieve a dark brown solution, from which the melanin NPs were collected via centrifugation at 5000 ​rpm and dried at 40 ​°C for 24 ​h.

***Preparation of the silk hydrogel*.** The silk hydrogel solution was prepared using silk cocoons (New Hope Silkworm Farm, Gokseong-gun, South Korea) that were cut into small pieces and boiled for 30 ​min in 0.02 ​M sodium carbonate (Sigma Aldrich) solution. Subsequently, degummed silk fibers were washed three times with distilled DI water. The obtained silk fibers were dried overnight to remove water. To dissolve the silk fibers, 1 ​g of CaCl_2_ (Sigma Aldrich) was first dissolved in 20 ​g formic acid (Daejung Chemicals), to which 3 ​g of dried silk fibers were added. Finally, 1.8 ​g of glycerol (Junsei Chemicals) was added as a plasticizer to obtain flexible and water resistible silk hydrogel layers.

***Printing photodetector arrays on Si*.** To prepare the 3D-printing bioink, 2 ​wt% of melanin NPs was dispersed in a silk hydrogel solution. The melanin NP-silk hydrogel ink was loaded into a syringe, which was then mounted in the 3D-printing machine. Si substrates were sequentially cleaned with acetone, propanol, and DI water and then placed in the printer. A nozzle size of 0.2 ​mm was used to make square shapes on the Si substrate. After printing, the samples were dried for 5 ​h.

***Device fabrication.*** The printed melanin NP-silk array on an Si substrate was stored for 2 ​h under 60% RH, after which a stripped ITO/glass substrate was placed over it to provide electrical contact. Finally, a Cu electrode was attached to the bottom of the Si substrate with Ag paste. For making a biocompatible device, AgNWs were integrated into a silk layer by following the method in our previous work.[39] Subsequently, instead of using an ITO/glass substrate, the AgNW-integrated silk layer was placed on the melanin NP-silk hydrogel array to fabricate a skin-attachable device.

***Cell Culture*.** HeLa cells were cultured in a cell medium, and silk layers were added to the medium. Before cell culture, the silk layers were cleaned with PBS and EtOH. The morphology of the layers was observed using an optical microscope on day 1 and day 4 of cell culture to assess the interaction of cells with the layer.

***Characterization*.** The printing process was performed by using a 3D printer (Rokit Invivo-3D bio-printer). The morphology and structure of the melanin NPs and the melanin NP-silk hydrogel layer were elucidated by using field-emission SEM (JSM-6700F, JEOL, Japan). The electrical measurements were studied using an HP4145B semiconductor parameter analyzer. A 532-nm laser and LEDs (365, 405, 617, 730 ​nm-Thorlab) were used for optical measurements.

## Declaration of competing interest

The authors declare that they have no known competing financial interests or personal relationships that could have appeared to influence the work reported in this paper.

## Data Availability

Data will be made available on request.
