# Supplementary material for: A biomaterial-silicon junction for photodetection
Source: Mater Today Bio. 2023 Apr 24;20:100642. doi: 10.1016/j.mtbio.2023.100642 (PMC10154958; doi:10.1016/j.mtbio.2023.100642)
Supplement: Multimedia component 1 [file mmc1.docx]

**A Biomaterial-Silicon Junction for Photodetection**

*Narendar Gogurla^1^, Abdul Wahab^1^, and Sunghwan Kim^2,*^*

^1^ *Department of Energy Systems Research, Ajou University, Suwon 16499, Korea*

*^2^ Department of Biomedical Engineering & Department of Electronic Engineering, Hanyang University, Seoul 04763, Korea*

*^*^ Corresponding Author: skim81@hanyang.ac.kr*

4 pages, 4 figures, S1-S4


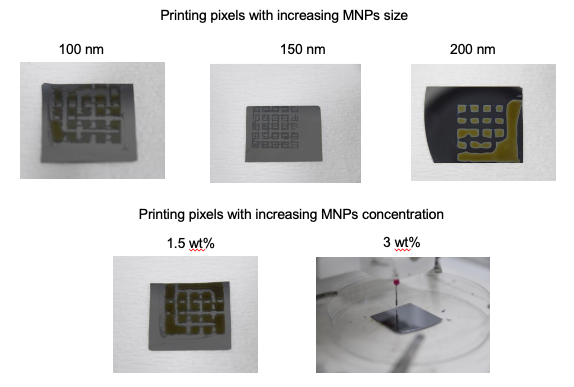


Figure S1: Printed pixels on Si with bioinks consisting of different (a) sizes and (b) concentration of melanin NPs in silk hydrogels.


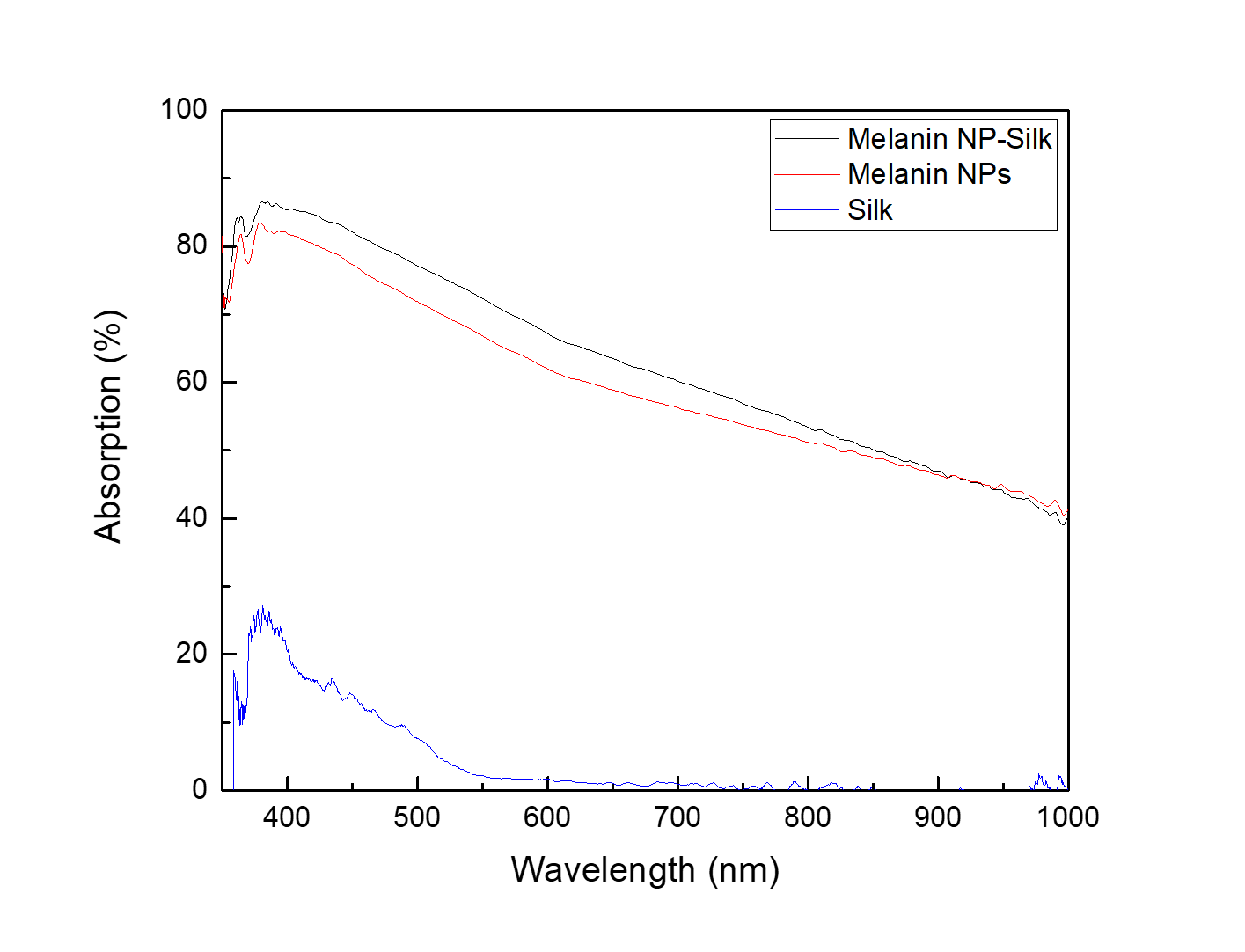


Figure S2: Absorption spectra of melanin NPs, silk hydrogel, and their composite.


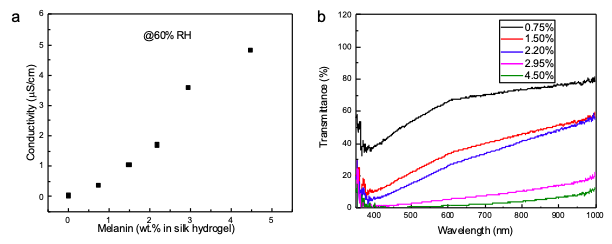


Figure S3: (a) Conductivities of the melanin NPs-silk hydrogel layers with increasing the concentration of melanin NPs. (b) Transmittance of the melanin NPs-silk layers at different concentrations of melanin NPs in silk hydrogel.


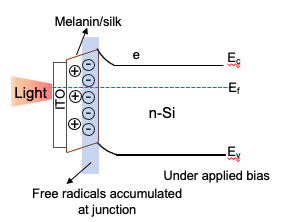


Figure S4: Schematic diagram to show the band structure and free radical accumulation at the junction in the melanin NP-silk/n-Si device.


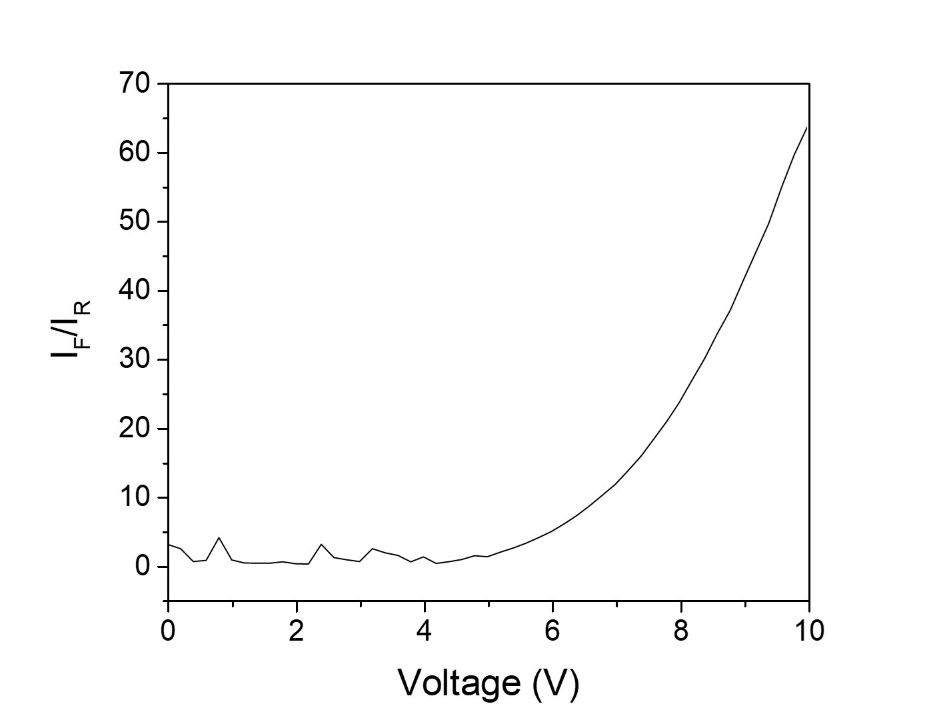


Figure S5: Rectification ratio of the device.


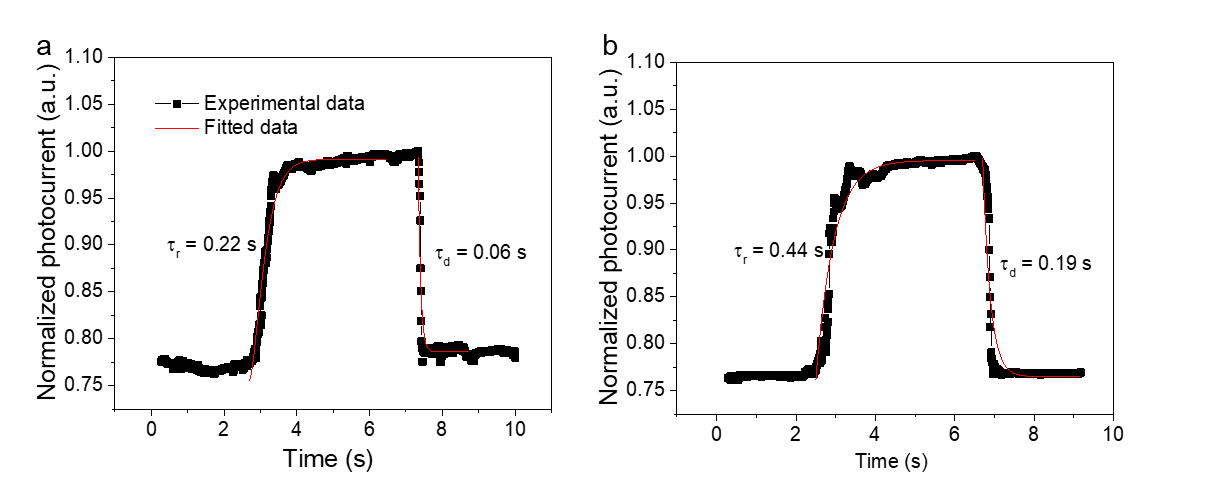


Figure S6: Fitting of transient photocurrents of (a) ITO/MNPs-silk/Si and (b) AgNWs/MNPs-silk/Si devices.


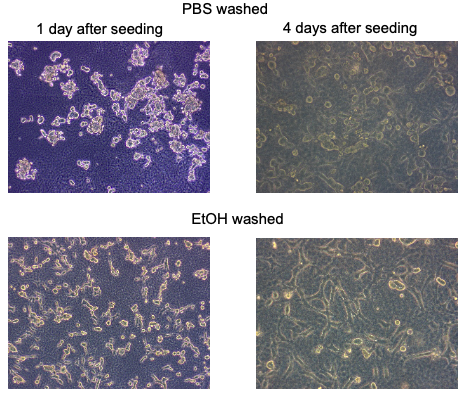


Figure S7: Photographs of cell cultured silk layer samples with seeded HeLa cells that are cleaned with PBS and EtOH.


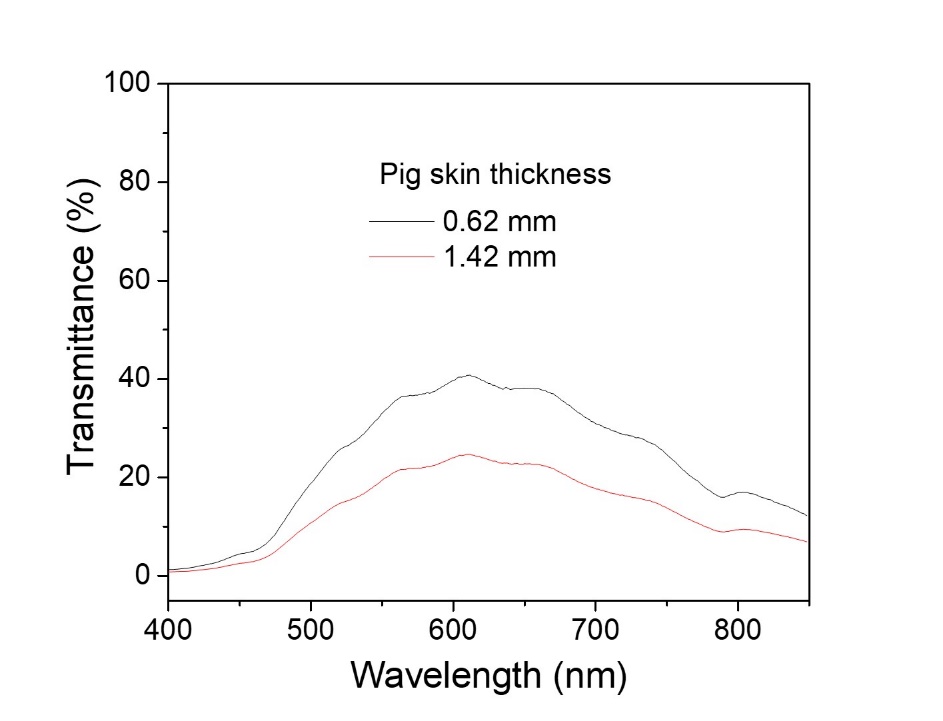


Figure S8: Transmittance spectra of the pig skin layers with different thicknesses.
